# Development of a New Semi-Selective Lysine-Ornithine-Mannitol-Arginine-Charcoal Medium for the Isolation of *Pantoea* Species from Environmental Sources in Japan

**DOI:** 10.1264/jsme2.ME18128

**Published:** 2019-03-28

**Authors:** Tatsuya Kuranishi, Jun-ichiro Sekiguchi, Izumi Yanagisawa, Makoto Akiwa, Yuko Tokuno

**Affiliations:** 1 Microbiology Research Division Kohjin Bio Co., Ltd. 5–1–3 Chiyoda, Sakado, Saitama 350–0214 Japan; 2 Department of Health and Nutrition, Faculty of Human Life, Jumonji University 2–1–28 Sugasawa, Niiza, Saitama 352–8510 Japan

**Keywords:** *Pantoea* sp., isolation, semi-selective medium, environment, phylogenetic analysis

## Abstract

Although *Pantoea* species are widely distributed among plants, water, soils, humans, and animals, due to a lack of efficient isolation methods, the clonality of *Pantoea* species is poorly characterized. Therefore, we developed a new semi-selective medium designated ‘lysine-ornithine-mannitol-arginine-charcoal’ (LOMAC) to isolate these species. In an inclusive and exclusive study examining 94 bacterial strains, all *Pantoea* strains exhibited yellow colonies on LOMAC medium. The performance of the medium was assessed using *Pantoea*-spiked soils. Percent average agreement relative to the Api20E biochemical test was 97%. A total of 24 soil spot samples and 19 plant types were subjected to practical trials. Of the 91 yellow colonies selected on LOMAC medium, 81 were correctly identified as *Pantoea* species using the biochemical test. The sequencing of 16S rRNA (*rrs*) and *gyrB* from these isolates confirmed that *Pantoea agglomerans*, *P. vagans*, *P. ananatis*, and *P. deleyi* were present in Japanese fields. A phylogenetic analysis using *rrs* enabled only the limited separation of strains within each *Pantoea* spp., whereas an analysis using *gyrB* revealed higher variability and enabled the finer resolution of distinct branches. *P. agglomerans* isolates were divided into 3 groups, 2 of which were new clades, with the other comprising a large group including biocontrol strains. *P. vagans* was also in one of the new clades. The present results indicate that LOMAC medium is useful for screening *Pantoea* species. The use of LOMAC medium will provide new opportunities for identifying the beneficial properties of Japanese *Pantoea* isolates.

*Pantoea* is a genus of Gram-negative bacteria of the family *Enterobacteriaceae*, recently separated from the genus *Enterobacter*. The genus *Pantoea* includes at least 20 species, such as *P. agglomerans*, *P. vagans* (formerly a *P. agglomerans* strain), *P. ananatis*, *P. deleyi*, and *P. eucalyptii* (25). Members of *Pantoea* are motile, non-encapsulated, non-spore forming rods with peritrichous flagella, and are typically yellow pigmented. *Pantoea* are abundant in plant and animal products, arthropods and other animals, water, soil, dust and air, and occasionally in humans. *Pantoea* species exhibit both deleterious and beneficial characteristics. For example, although *Pantoea* species are known to cause crop diseases and disorders in exposed individuals via the inhalation of organic dusts (5–7, 16, 18), they also produce substances effective in the treatment of various cancers in humans and animals, suppress the development of various plant pathogens via antibiotic production and/or competition, and exhibit bio-fertilizer and bio-remediation properties (6).

Previous studies reported the isolation of unique *Pantoea* strains from environmental sources. Son *et al*. in Korea, Malboobi *et al*. in Iran, and Sulbaran *et al*. in Venezuela demonstrated that *P. agglomerans* strains isolated from soil exert beneficial effects on crops (6, 14, 15, 23, 24). In greenhouse and field trials, Malboobi *et al*. showed that *P. agglomerans* promoted the growth of potato plants (14, 15). Kageyama *et al*. isolated new *Pantoea* spp. from fruits and soils in Japan and demonstrated that these Japanese species were phylogenetically distant from other *Pantoea* species (3, 11). Japanese researchers also reported the efficacy of LPS from *P. agglomerans* for the treatment of human cancers. Kasugai *et al*. intradermally administered LPS in combination with transarterial intermittent chemotherapy to treat patients with advanced gastric cancer with multiple liver metastases (12).

Despite the apparent medical and agricultural significance of *Pantoea* spp., limited information is currently available on their distribution and prevalence, which is partly due to the lack of methods for efficient isolation and enumeration in the presence of competing organisms. Only a few media for detecting *Pantoea* spp. have been reported to date. Lysine-Ornithine-Mannitol (LOM) agar was developed in 1981 for isolating *Enterobacter agglomerans* (4); however, this medium was developed for testing human stool samples. PA 20 semi-selective medium was developed in 2006 for the isolation and enumeration of *P. ananatis* from plant material (10). Non-selective agar media, such as Nutrient agar and LB, are occasionally used to isolate *Pantoea* spp. The ability to produce a yellow pigment is used to identify *Pantoea* spp. on non-selective agar media, such as LB and Trypticase soy agar (5, 8, 9). However, the detection and isolation of *Pantoea* spp. from environmental sources (1, 9) will require a new semi-selective agar medium. Therefore, the purpose of the present study was to develop a semi-selective medium for the isolation and enumeration of *Pantoea* spp. in the presence of competing organisms frequently found in soils and plants.

## Materials and Methods

### Semi-selective agar medium

The ingredients of lysine-ornithine-mannitol-arginine-charcoal (LOMAC) agar medium were placed into two groups. Solution A was prepared by adding the following (L^−1^) to water: 3 g of yeast extract (Difco Laboratories, Detroit, MI, USA), 2 g of sodium chloride (Wako Pure Chemical Industries, Osaka, Japan), 2 g of magnesium sulfate (Wako Pure Chemical Industries), 0.05 g of sodium pyruvate (Wako Pure Chemical Industries), 1 g of soy peptone (Conda, Madrid, Spain), 5 g of L-lysine hydrochloride (Peptide Institute, Osaka, Japan), 6.5 g of L(+)-ornithine hydrochloride (Peptide Institute), 5 g of L-arginine hydrochloride (Peptide Institute), 0.3 g of bromothymol blue (Sigma Aldrich, St. Louis, MO, USA), 13.5 g of agar (SSK Sales, Shizuoka, Japan), and 2 g of charcoal (Serachem, Hiroshima, Japan). pH was adjusted to 6.5. Solution A was autoclaved at 121°C for 15 min and cooled to 50°C. Solution B was prepared by adding the following (50 mL^−1^) to water: 5.25 g of mannitol (Wako Pure Chemical Industries), 0.03 g of vancomycin hydrochloride (Wako Pure Chemical Industries), and 0.016 g of amphotericin B (Wako Pure Chemical Industries). Fifty milliliters of solution B was filter-sterilized and then mixed with 1 L of solution A. Charcoal was used as an absorbent against toxic chemicals to bacteria. Vancomycin and amphotericin B were used as inhibitors of Gram-positive bacteria and fungi, respectively. Media were designed for mannitol-positive, lysine-negative, ornithine-negative, and arginine-negative species, including *Pantoea* spp., to yield intensely yellow colonies (8). Mannitol-, lysine-, ornithine-, and arginine-negative species yield colorless colonies. Species that are mannitol-positive and either lysine-, ornithine-, or arginine-positive yield greenish-blue colonies. Species that are mannitol-negative and either lysine-, ornithine-, or arginine-positive yield colorless colonies that turn greenish-blue after more than 24 h.

### Plating efficiency tests

A total of 86 bacterial and 8 fungal strains were examined (Table 1). These included 69 Gram-negative rods, including 13 *Pantoea* spp., 14 Gram-positive cocci, and 3 Gram-positive rods. These strains were sub-cultured on non-selective medium (Tryptic Soy Agar [TSA]; Difco Laboratories) at 35±2°C for 24 h. An overnight TSA culture of each bacterial colony was streaked onto LOMAC and LOM plates (4), which were incubated at 35±2°C and examined after 24 h for the presence or absence of yellow colonies.

The recovery of *Pantoea* species using LOMAC medium was evaluated based on the efficiency of colony formation. Bacterial suspensions from fresh colonies grown on TSA were adjusted in sterile saline to an optical density at 660 nm (OD_660_) of 0.5 (*ca*. 1.5×10^8^ CFU mL^−1^). Bacterial suspensions were serially diluted 10-fold, and 0.1-mL aliquots of each dilution were plated on the tested plate media. The recovery percentage was calculated from the ratio of the mean colony counts on the test medium and on non-selective TSA as a reference.

### Verification of LOMAC agar medium for the cultivation of *Pantoea* spp. from environmental soils

The effectiveness of the LOMAC plate for bacterial recovery was demonstrated in soils (1 g) spiked with 1:10^5^ dilutions of *P. agglomerans* NBRC 102470 (1.5×10^8^ CFU mL^−1^). Soils spiked with *P. agglomerans* were added to sterile saline (20 mL) and vortexed for 30 s in a mixer. The supernatant was serially diluted 1:10 in sterile saline, and 0.1 mL of the suspension was spread on LOMAC agar. The plates were incubated for 24 to 48 h at 35±2°C. After the incubation, all colonies obtained were streaked onto TSA medium and incubated at 35±2°C for 24 h. Isolates were identified by biochemical testing using the Api20E system (Sysmex bioMérieux, Tokyo, Japan).

### Sample collection and practical trials

Samples of plants and environmental soils were obtained from 26 areas in Japan. Samples of soil (1 g) were added to sterile saline (20 mL) and vortexed for 30 s in a mixer. Similarly, plant samples (1 g) were added to sterile saline (10 mL) and homogenized using a mortar and pestle. After filtering using a cell strainer (40 μm), the flow through fraction was serially diluted 1:10 in sterile saline, and 0.1 mL of the suspension was spread on LOMAC agar medium and non-selective TSA medium. After an incubation at 35±2°C for 24 to 48 h, bacterial colonies with a yellow color were selected, passaged onto TSA medium, and incubated at 35±2°C for 24 h. After a second incubation, the isolates were identified by biochemical testing using the Api20E system.

### PCR and phylogenetic analysis of sequencing data

Bacterial DNA was extracted using a QIAamp UCP Pathogen Mini kit (Qiagen KK, Tokyo, Japan). PCR amplification of the housekeeping genes 16S rRNA (*rrs*) and *gyrB* was performed using the following primer sets: 16S-8F and 16S-1492R for *rrs*, and gyr-320 and rgyr-1260 for *gyrB* (20). PCR targeting for *rrs* encoding 16S rRNA was performed with the ExTaq (Takara Bio, Shiga, Japan) enzyme under the same conditions as those previously described (20), except for an annealing temperature set to 49°C. PCR targeting for *gyrB* encoding partial GyrB was performed with initial denaturation and activation of the ExTaq enzyme at 95°C for 5 min, followed by 35 cycles of denaturation at 94°C for 30 s, annealing at 50°C for 45 s, elongation at 72°C for 60 s, and final elongation at 72°C for 7 min. Positive PCR amplification was verified electrophoretically using 5 μL of each reaction loaded onto a 1.5% agarose gel. PCR products were verified by DNA sequencing. Briefly, the PCR amplicon was purified with the NucleoSpin Gel and PCR Clean-up kit (MACHEREY-NAGEL GmbH & Co. KG, Duren, Germany) and subjected to DNA sequencing. In 16S rRNA sequencing, additional primers, 16S-609R and 16S-533R, were used to achieve the complete coverage of the amplicon (20). In GyrB sequencing, DNA sequences were elucidated by the dideoxy termination method employing the same primers used for PCR amplification. Nucleotide sequences were searched for homology by BLAST screening against the GenBank databases. DNA sequences were aligned using ClustalW, and phylogenetic trees were generated based on partial *gyrB* sequences. Sites exhibiting alignment gaps were excluded from the analysis. NJplot program (19), version 2.3, was used to calculate evolutionary distances and infer trees based on the minimum evolution (ME) method using the maximum composite likelihood formula. The nodal robustness of the inferred trees was assessed using 1,000 bootstrap replicates.

### Nucleotide sequence accession number

The nucleotide sequences of the isolates in Japan reported here have been deposited in the EMBL/GenBank/DDBJ databases and assigned the following accession numbers: LC422596 and LC422697 to LC422728 for the nucleotide sequence of *gyrB*; LC438406 to LC438434 for the nucleotide sequence of *rrs*.

## Results

### Growth and selectivity tests

To assess the selectivity of LOMAC medium for *Pantoea* strains and the growth of these strains on the medium, 86 bacterial strains, including *P. agglomerans* NBRC 102470, 6 isolates of *P. agglomerans*, 2 isolates of *P. ananatis*, *P. brenneri* ES153, *P. deleyi* ES168, 2 isolates of *P. vagans*, and 8 fungal strains were streaked on agar plates (Table 1). All *Pantoea* strains tested formed yellow colonies on LOMAC medium after being incubated for 24 h. However, 5 Gram-negative rods also formed yellow colonies on LOMAC medium. *Acinetobacter lwoffii* 85, *Candida kefyr* 116, *Cryptococcus neoformans* 105, and 14 Gram-positive cocci and 3 Gram-positive rods did not grow on the medium. The remaining isolates, including 50 Gram-negative rods and 6 fungi, formed colonies of various colors, such as blue, green, blue-white, white, whitish-yellow, clear, brownish-yellow, and red, but did not form yellow colonies. These results indicated that LOMAC medium is not only semi-selective for *Pantoea* strains, but also enables the differentiation of strains based on colony color.

Similarly, the selectivity of LOM medium for *Pantoea* strains and growth of the strains on the medium were tested (Table 1). All *Pantoea* strains tested formed yellow colonies on LOM medium after being incubated for 24 h. However, 30 Gram-negative rods and 2 fungi also formed yellow colonies on LOM medium. The remaining isolates, including 25 Gram-negative rods and 4 fungi, formed colonies of various colors, but did not form yellow colonies. There were no strains that were present on LOMAC medium but not on LOM medium. These results indicated that LOM medium is equal to LOMAC medium for selectivity to *Pantoea* strains, but not superior to LOMAC for the differentiation of strains based on colony color.

### Recovery of growth on LOMAC medium

LOMAC medium and TSA medium were compared in terms of recovering the growth of 6 *P. agglomerans* strains, 2 *P. ananatis* strains, one *P. brenneri* strain, one *P. deleyi* strain, and 2 *P. vagans* strains. Plating efficiencies for the 12 *Pantoea* strains ranged between 44 and 182, *i.e*., 106% for *P. agglomerans* ES53, 92% for *P. agglomerans* ES127, 78% for *P. agglomerans* ES137, 56% for *P. agglomerans* ES144, 94% for *P. agglomerans* ES149, 93% for *P. agglomerans* ES162, 44% for *P. ananatis* ES126, 70% for *P. ananatis* ES133, 112% for *P. brenneri* ES153, 182% for *P. deleyi* ES168, 143% for *P. vagans* ES63, and 159% for *P. vagans* ES 67, with an average of 102%. The colonies of all *Pantoea* strains were yellow, convex, with smooth margins, and visible after the incubation at 35±2°C for 24 h. These results indicated that LOMAC medium supported the good growth of the *Pantoea* strains tested.

### Evaluation of LOMAC agar medium for the cultivation of *Pantoea* spp. in soils

Bacterial recovery from soils was assessed using 3 different soils spiked with *P. agglomerans* NBRC 102470 (Table 2). LOMAC agar plates enabled the growth of 99 colonies, 10 of which were colonies with a yellow color. Fifty-five colonies were obtained from soil A; five of these were colonies with a yellow color and correctly identified as *Pantoea* spp.3 using Api20E. The other 50 colonies with non-yellow colors were not identified as any *Pantoea* species (Table 2). Soil B yielded 4 colonies with a yellow color. Only 1 colony was identified as *Pantoea* spp.3. The other 2 colonies were identified as *Citrobacter youngae* and the remaining colony as *Leclercia adecarboxylate*. Soil B also yielded 26 colonies with non-yellow colors. None of them were identified as *Pantoea* species. Similarly, Soil C yielded 1 colony with a yellow color, which was identified as *Pantoea* spp. 3. Soil C yielded 13 colonies with non-yellow colors, none of which were identified as *Pantoea* species (Table 2). Average percent positive and negative predictive values were 70% (7/10) and 100% (89/89), respectively. Overall agreement was 97% (96/99), indicating that *Pantoea* species were successfully isolated on the medium and that the colonies with a yellow color were instantaneously distinguishable as *Pantoea* species.

### Sample collection and practical trials for isolating *Pantoea* species

**Fig. 1:**
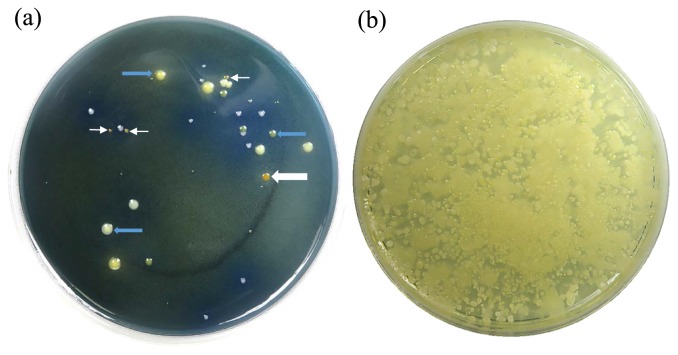
(a) Bacterial colonies on lysine-ornithine-mannitol-arginine-charcoal (LOMAC) agar medium. (b) Bacterial colonies on Tryptic soy agar medium. White arrows indicate bacterial colonies actually defined as yellow. Blue arrows indicate bacterial colonies defined as other than yellow. Samples were equally applied to each medium.

A total of 26 trials for isolating *Pantoea* species were performed using 24 spots of soil samples and 19 samples of plants obtained from geographically diverse regions of Japan, such as Nagano, Fukuoka, Chiba, and Hokkaido (Table 3). All samples were tested with LOMAC and TSA. LOMAC agar typically generated yellow colonies (Fig. 1a), whereas TSA medium did not have the ability to isolate *Pantoea* species producing a yellow pigment (Fig. 1b).

A total of 797 yellow colonies were generated on LOMAC agar (Table 3). Among these colonies, 91 were sub-cultured on TSA medium. Eighty-one out of the 91 colonies were identified as *Pantoea* spp. using the Api20E test. One isolate was identified as *Pantoea* spp. 1, 18 as *Pantoea* spp. 2, 61 as *Pantoea* spp. 3, and 1 as *Pantoea* spp. 4. These results demonstrated that LOMAC agar is applicable for use in the isolation of various *Pantoea* species, such as *Pantoea* spp. 1, spp. 2, spp. 3, and spp. 4.

### PCR and sequencing results

In further analyses of the 81 *Pantoea* spp. colonies, 34 genomic DNAs were randomly selected and subjected to PCR amplification targeting the *rrs* gene. In 29 out of 34 genomic DNAs, PCR analyses yielded 1503-bp amplicons of the expected size.

DNA sequence analyses revealed that all of the sequences of the 20 *Pantoea* spp. 3 isolates were 100% identical not only to one another, but also some types of *Pantoea* strains, such as *P. agglomerans* ATCC27155, *P. deleyi* LMG24200 (20), and *P. vagans* LMG24199 (20); the *rrs* sequences of 8 isolates of *Pantoea* spp. 2 were categorized into 3 types of sequences for their highest identity, *i.e*., the sequences of the *rrs* of 5 isolates of *Pantoea* spp. 2 were 100% identical to *P. ananatis* ATCC 27966 [*P. ananatis* type]; the sequences of 2 *Pantoea* spp. 2 isolates showed the highest identity to that of *Erwinia aphidicola* ATCC 27992 (ranging from 99% to 100% nucleotide [nt] identity) (*Erwinia* type); the sequence of one *Pantoea* spp. 2 isolate was 100% identical to *P. agglomerans* ATCC 27155 (*P. agglomerans* type); and the sequence of one *Pantoea* spp. 4 isolate showed the highest identity to that of *Enterobacter cloacae* ATCC 13047 (99% nt identity) (*E. cloacae* type).

In all 34 genomic DNAs, PCR targeting the *gyrB* gene yielded 970-bp amplicons of the expected size. DNA sequence analyses and a BLAST search of the *gyrB* amplicons revealed that the *gyrB* sequence of *Pantoea* spp. 1 isolate was nearly identical to that of *E. toletana* (85% nucleotide [nt] identity); the *gyrB* sequences of 9 isolates of *Pantoea* spp. 2 were categorized into 3 types of sequences for their highest identity, *i.e*., the sequence of one isolate of *Pantoea* spp. 2 showed the highest identity to that of *P. vagans* (97% nt identity) (*P. vagans* type), the sequences of 6 isolates of *Pantoea* spp. 2 showed the highest identity to that of *P. ananatis* (ranging between 91 and 100% nt identity) (*P. ananatis* type); the sequence of one isolate of *Pantoea* spp. 2 showed the highest identity to that of *E. rhapontici* (90% nt identity) (*Erwinia* type), and the sequence of one isolate of *Pantoea* spp. 2 showed the highest identity to that of *E. tasmaniensis* (87% nt identity) (*Erwinia* type); the *gyrB* sequences of 23 isolates of *Pantoea* spp. 3 were categorized into 4 types of sequences for their highest identity, *i.e*., the sequences of 15 isolates of *Pantoea* spp. 3 showed the highest identity to that of *P. agglomerans* (ranging between 95 and 99% nt identity) (*P. agglomerans* type), the sequences of 5 isolates of *Pantoea* spp. 3 showed the highest identity to that of *P. vagans* (ranging between 97 and 100% nt identity) (*P. vagans* type), the sequences of 2 isolates of *Pantoea* spp. 3 showed the highest identity to that of *P. deleyi* (99% nt identity) (*P. deleyi* type), and the sequence of one isolate of *Pantoea* spp. 3 showed the highest identity to that of *P. brenneri* (98% nt identity) (*P. brenneri* type); and the sequence of one isolate of *Pantoea* spp. 4 showed the highest identity to that of *L. adecarboxylata* (95% nt identity).

### Phylogeny of *Pantoea* isolates obtained in practical trials

**Fig. 2:**
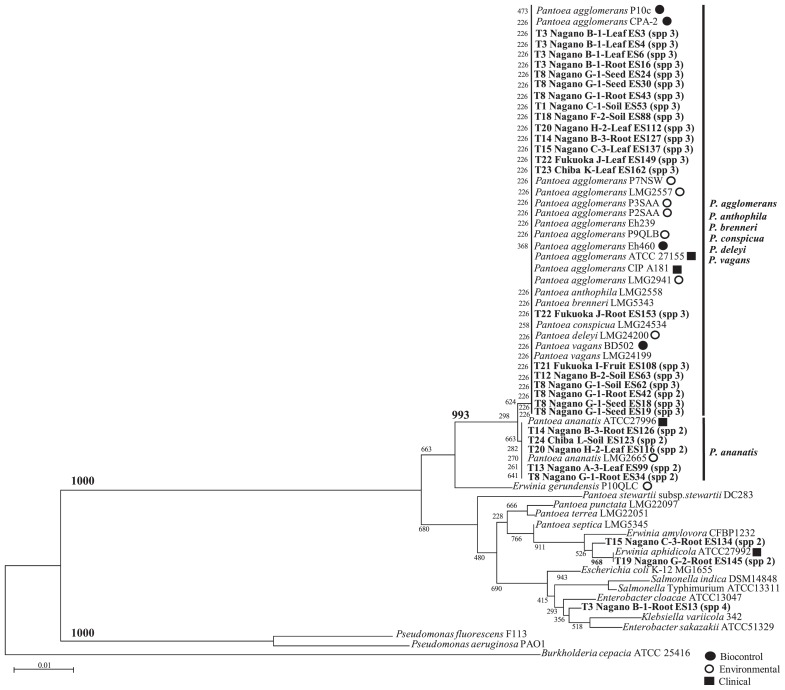
Phylogenetic relationships based on 16S ribosomal DNA sequences obtained from strains related to *Pantoea* spp. isolated from soil and plant samples in isolation practical trials. The dendrogram was generated using the neighbor-joining method. The numbers on the branches represent confidence intervals generated by bootstrapping with 1,000 replications. The scale bar represents 0.1 substitutions per nucleotide position. The nucleotide sequences of the isolates in Japan reported herein have been deposited in the EMBL/GenBank/DDBJ databases and assigned accession numbers. The accession numbers of the referenced strains are as follows: *P. agglomerans* CIP181 (GenBank: FJ611803.1), *P. agglomerans* P7NSW (GenBank: FJ611823.1), *P. agglomerans* CPA-2 (GenBank: FJ611834.1), *P. agglomerans* P2SAA (GenBank: FJ611819.1), *P. agglomerans* P10c (GenBank: FJ611836.1), *P. agglomerans* LMG2557 (GenBank: FJ611802.1), *P. agglomerans* Eh239 (GenBank: FJ611826.1), *P. agglomerans* P3SAA (GenBank: FJ611820.1), *P. agglomerans* Eh460 (GenBank: FJ611828.1), *P. agglomerans* P9QLB (GenBank: FJ611831.1), *P. agglomerans* LMG2941 (GenBank: FJ611837.1), *P. conspicua* LMG24534 (GenBank: NR_116247.1), *P. brenneri* LMG5343 (GenBank: NR_116748.1), *P. vagans* BD502 (GenBank: DQ849043.1), *P. vagans* LMG24199 (GenBank: NR_116115.1), *P. anthophila* LMG2558 (GenBank: NR_116113.1), *P. deleyi* LMG24200 (GenBank: NR_116114.1), *Salmonella enterica subsp. indica* DSM14848 (GenBank: NR_044370.1), *S. enterica subsp. enterica* serovar Typhimurium ATCC13311 (GenBank: NR_119108.1), *Enterobacter cloacae* ATCC13047 (GenBank: NR_102794.2), *E. sakazakii* ATCC51329 (GenBank: GU122217.1), *P. stewartii subsp. stewartii* DC283 (GenBank: AJ311838.1), *P. ananatis* LMG2665 (GenBank: NR_119362.1), *P. ananatis* ATCC27996 (GenBank: FJ611813.1), *P. septica* LMG5345 (GenBank: NR_116752.1), *E. gerundensis* P10QLC (GenBank: FJ611850.1), *E. aphidicola* ATCC27992 (GenBank: FJ611858.1), *E. amylovora* CFBP1232 (GenBank: NR_116753.1), *P. punctate* LMG22097 (Japanese *Pantoea*) (GenBank: FJ611886.1), *P. terrea* LMG22051 (Japanese *Pantoea*) (GenBank: NR_116110.1), *Escherichia coli* str. K-12 substr. MG1655 (Genbank: U00096.3), *Burkholderia cepacia* ATCC 25416 (Genbank: AF097530.1), *Pseudomonas aeruginosa* PAO1 (Genbank: DQ777865.1), *P. fluorescens* F113 (Genbank: CP003150.1), *Klebsiella variicola* 342 (Genbank: CP000964.1).

A dendrogram was calculated using the partial *rrs* sequences of a length appropriate for the analysis (Fig. 2). A total of 29 *rrs* sequences were obtained in the present study. Based on the results of phylogenetic tree analyses on 29 strains, the *Pantoea* strains isolated in the present study were roughly divided into 2 groups. The first group mainly consisted of *Pantoea* species, including *P. agglomerans*, *P. anthophila*, *P. brenneri*, *P. deleyi*, *P. vagans*, and *P. ananatis*. The second group consisted of *P. stewartii*, *P. terrea*, *P. septica*, *P. punctata*, and other *Enterobacteriaceae* (Fig. 2). Analyses using 16S rDNA enabled only the limited separation of strains within each *Pantoea* spp.

**Fig. 3:**
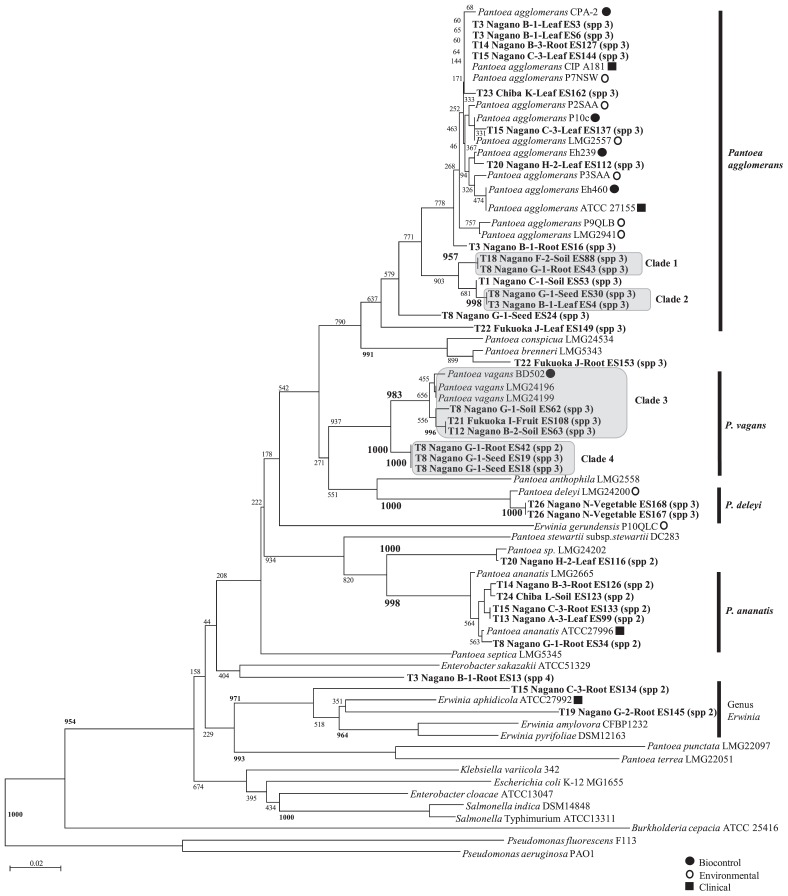
Phylogenetic relationships based on partial *gyrB* sequences obtained from strains related to *Pantoea* spp. isolated from soil and plant samples in isolation practical trials. The dendrogram was generated using the neighbor-joining method. The numbers on the branches represent confidence intervals generated by bootstrapping with 1,000 replications. The scale bar represents 0.1 substitutions per nucleotide position. The nucleotide sequences of the isolates in Japan reported herein have been deposited in the EMBL/GenBank/DDBJ databases and assigned accession numbers. The accession numbers of the referenced strains are as follows: *P. agglomerans* CIP181 (GenBank: FJ617394.1), *P. agglomerans* P7NSW (GenBank: FJ617397.1), *P. agglomerans* CPA-2 (GenBank: FJ617395.1), *P. agglomerans* P2SAA (GenBank: FJ617388.1), *P. agglomerans* P10c (GenBank: FJ617389.1), *P. agglomerans* LMG2557 (GenBank: FJ617408.1), *P. agglomerans* Eh239 (GenBank: FJ617379.1), *P. agglomerans* P3SAA (GenBank: FJ617392.1), *P. agglomerans* Eh460 (GenBank: FJ617377.1), *P. agglomerans* P9QLB (GenBank: FJ617403.1), *P. agglomerans* LMG2941 (GenBank: FJ617399.1), *P. conspicua* LMG24534 (GenBank: EU145269.1), *P. brenneri* LMG5343 (GenBank: FJ617409.1), *P. vagans* BD502 (GenBank: EF988786.1), *P. vagans* LMG24196 (GenBank: EF988758.1), *P. vagans* LMG24199 (GenBank: EF988768.1), *P. anthophila* LMG2558 (GenBank: EF988812.1), *P. deleyi* LMG24200 (GenBank: EF988770.1), *Salmonella enterica subsp. indica* DSM14848 (GenBank: EU014648.1), *S. enterica subsp. enterica* serovar Typhimurium ATCC13311 (GenBank: EU014643.1), *Enterobacter cloacae* ATCC13047 (GenBank: EU643470.1), *E. sakazakii* ATCC51329 (GenBank: AY370844.1), *P. stewartii subsp. stewartii* DC283 (GenBank: KF482574.1), *Pantoea sp*. LMG24202 (GenBank: EF988778.1), *P. ananatis* LMG2665 (GenBank: KF482590.1), *P. ananatis* ATCC27996 (GenBank: FJ617369.1), *P. septica* LMG5345 (GenBank: FJ617422.1), *E. gerundensis* P10QLC (GenBank: FJ617414.1), *E. aphidicola* ATCC27992 (GenBank: FJ617417.1), *E. amylovora* CFBP1232 (GenBank: FJ617419.1), *E. pyrifoliae* DSM12163 (GenBank: AB242885.1), *P. punctate* LMG22097 (Japanese *Pantoea*) (GenBank: EF988805.1), *P. terrea* LMG22051 (Japanese *Pantoea*) (GenBank: EF988804.1), *Escherichia coli* str. K-12 substr. MG1655 (Genbank: NC_000913.3), *Burkholderia cepacia* ATCC 25416 (Genbank: HQ849191.1), *Pseudomonas aeruginosa* PAO1 (Genbank: AE004091.2), *P. fluorescens* F113 (Genbank: CP003150.1), *Klebsiella variicola* 342 (Genbank: CP000964).

A total of 33 *gyrB* sequences were obtained. Based on the results of phylogenetic tree analyses for 33 strains, the *Pantoea* strains isolated in the present study were roughly divided into a number of groups (Fig. 3). In addition, *P. agglomerans* was further divided into three clades; clades 1 and 2 did not contain previously reported strains. The other clade formed a large group containing the *P. agglomerans*-type strain and already reported biocontrol strains (20). Similarly, *P. vagans* was also divided into two clades, in which clade 3 contained strain BD502, which was previously reported as a biocontrol strain (20), and clade 4 did not contain any previously reported strains. *P. deleyi* grouped with strain LMG24200, which was previously reported as an environmental strain (2). *P. ananatis* formed a large group in which all of the isolates were found to belong to *Pantoea* spp. 2. These results indicated that LOMAC medium is applicable to the isolation of *Pantoea* species exhibiting novel phylogenetic properties.

## Discussion

We herein developed a new semi-selective agar medium and proposed a protocol for isolating *Pantoea* species. On LOMAC medium, *Pantoea* strains formed yellow colonies; however, some Gram-negative bacteria from environmental samples also formed yellow colonies. It is conceivable that under the same substrate availability conditions, strains other than *Pantoea* will form colonies of the same color. However, many *Pantoea* strains are known to produce a yellow pigment (5, 8). When yellow colonies isolated on LOMAC medium were passaged on TSA medium, the majority of *Pantoea* strains in the present study produced a yellow pigment on TSA. This result indicates that the diagnostic accuracy of the procedure may be improved by eliminating bacteria that do not produce a yellow pigment on TSA. The efficient recovery of *Pantoea* strains on LOMAC medium suggests its applicability to investigations on the ecology of these species in the environment.

In practical trials, many *Pantoea* strains were isolated from plants. *Pantoea* is a plant-derived bacterium known to exist as an endophyte. As reported previously (21, 22), the inside of plants is considered to be suitable for the survival of *Pantoea* species, such as *P. vagans*. Endophytic organisms, such as *Pantoea*, live inside plants without causing damage (17). In addition, endophytic *Pantoea* may promote plant growth by accelerating processes including nitrogen fixation, phosphate solubilization, siderophore secretion, and biocontrol (13, 17). Different *Pantoea* species were detected in different parts of the same plant in the present study. For example, 6 strains of *P. ananatis* were isolated from the roots of crops (dicot) in trial 8, but were not detected in seeds. This result suggests that the role of parasitism differs among species and also that each *Pantoea* species may play a different role inside plants. Notably, *P. deleyi* was detected in trial 26 in the stem of a vegetable plant. Although limited information is currently available on *P. deleyi*, this species has been isolated from bacterial plaques and dead portions of eucalyptus (2). However, there were no dead portions in the stems of the vegetables examined in this trial, and, therefore, the function of *P. deleyi* in these plants remains unclear. This species is predicted to be more closely related to *P. vagans* based on the results of *gyrB* phylogenetic tree analyses. Future studies will provide more information on this strain.

*Pantoea* strains were also isolated from 4 out of 24 spots of soil samples (16.7%) and from 15 out of 19 plant types (78.9%) with a large difference in detection rates. The population of *Pantoea* strains varies among crops, weeds, vegetables, fruits and soils in the environment in Japan.

Attempts to isolate *Pantoea* strains in 11 trials using samples from Nagano and 1 trial using samples from Hokkaido were unsuccessful because no colonies with a yellow color were observed on LOMAC medium. Among the non-yellow colonies, 7 were randomly selected and subjected to genetic analyses. As expected, *gyrB* sequencing revealed that these 7 colonies were not *Pantoea* species. In consideration of this result as well as recovery efficiency from soils, the protocol used in the present study is suitable for isolating *Pantoea* species.

In conclusion, we herein developed a new semi-selective medium known as LOMAC and established a protocol for isolating *Pantoea* species with high test efficiency. We detected *Pantoea* strains in samples of plants and soils from Japan using LOMAC even when *Pantoea* species were present at lower densities than non-target bacteria. Therefore, LOMAC medium enables the screening of *Pantoea* species from environmental sources and may be useful in future studies.

## Figures and Tables

**Table 1:** Growth and colony color of microbes cultivated on semi-selective agar media LOMAC agar and LOM agar

Nomenclature	Bacterial strains	Colony color	

		LOMAC*2	LOM
Gram-negative rod	*Pantoea agglomerans* NBRC 102470*1	**Yellow**	**Yellow**
	*Pantoea agglomerans* ES53	**Yellow**	**Yellow**
	*Pantoea agglomerans* ES127	**Yellow**	**Yellow**
	*Pantoea agglomerans* ES137	**Yellow**	**Yellow**
	*Pantoea agglomerans* ES144	**Yellow**	**Yellow**
	*Pantoea agglomerans* ES149	**Yellow**	**Yellow**
	*Pantoea agglomerans* ES162	**Yellow**	**Yellow**
	*Pantoea ananatis* ES126	**Yellow**	**Yellow**
	*Pantoea ananatis* ES133	**Yellow**	**Yellow**
	*Pantoea brenneri* ES153	**Yellow**	**Yellow**
	*Pantoea deleyi* ES168	**Yellow**	**Yellow**
	*Pantoea vagans* ES63	**Yellow**	**Yellow**
	*Pantoea vagans* ES67	**Yellow**	**Yellow**
	*Acinetobacter baumannii* 142	Blue	Brownish yellow
	*Acinetobacter baumannii* 610	Green	Brownish yellow
	*Acinetobacter lwoffii* 85	—*3	—
	*Cedecea davisae* 369	Blue white	Green
	*Cedecea davisae* 1319	Blue white	**Yellow**
	*Citrobacter freundii* 370	**Yellow**	**Yellow**
	*Citrobacter freundii* 711	White	**Yellow**
	*Edwardsiella trada* 371	Blue white	Blue white
	*Enterobacter aerogenes* 373	Blue white	Brownish yellow
	*Enterobacter aerogenes* 1278	Blue white	Brown
	*Enterobacter amnigenus* 374	White	**Yellow**
	*Enterobacter asburiae* 375	Blue	**Yellow**
	*Enterobacter cloacae* 87	Blue	Brown
	*Enterobacter cloacae* 372	Blue white	**Yellow**
	*Enterobacter cloacae* 726	Blue white	Whitish yellow
	*Enterobacter intermedius* 378	Blue	**Yellow**
	*Enterobacter kobei* 379	Blue white	**Yellow**
	*Enterobacter sakazakii* 380	Green	**Yellow**
	*Escherichia coli* 84	Blue white	**Yellow**
	*Escherichia coli* 381	White	**Yellow**
	*Escherichia coli* 722	White	Brownish yellow
	*Hafnia alvei* 382	Blue	Brownish yellow
	*Klebsiella oxytoca* 500	Blue white	**Yellow**
	*Klebsiella oxytoca* 1248	Blue white	**Yellow**
	*Klebsiella pneumoniae* 54	Blue	Brown
	*Klebsiella pneumoniae subsp. Ozaenae* 1233	Blue	Brown
	*Klebsiella Pneumoniae subsp. pneumoniae* 383	Whitish yellow	Brownish yellow
	*Klebsiella pneumoniae subsp. pneumoniae* 1223	**Yellow**	Brown
	*Klebsiella pneumoniae* 476	Blue white	Brown
	*Morganella morganii* 1434	Blue	**Yellow**
	*Plesiomonas shigelloides* 73	Blue white	Blue
	*Proteus mirabilis* 81	White	**Yellow**
	*Proteus vulgaris* 56	**Yellow**	**Yellow**
	*Proteus vulgaris* 384	White	**Yellow**
	*Providencia alcalifaciens* 1454	Whitish yellow	**Yellow**
	*Providencia rettgeri* 385	Clear	**Yellow**
	*Pseudomonas aeruginosa* 729	White	Brownish yellow
	*Pseudomonas fluorescens* 607	Blue white	Brown
	*Shewanella putrefaciens* 147	Blue	Brown
	*Vibrio alginolyticus* 150	Brownish yellow	Brown
	*Vibrio fluvialis* 153	Blue green	**Yellow**
	*Vibrio mimicus* 151	Blue green	Green
	*Vibrio vulnificus* 149	Green	Green
	*Yersinia enterocolitica* 134	**Yellow**	**Yellow**
	*Yersinia enterocolitica* 389	Clear	**Yellow**
	*Salmonella typhimurium* 677	Blue	Brownish yellow
	*Salmonella enteritidis* 672	Blue	**Yellow**
	*Shigella flexneri* 80	Blue	**Yellow**
	*Shigella sonnei* 143	Blue	**Yellow**
	*Serratia ficaria* 1364	Blue	**Yellow**
	*Serratia fonticola* 1369	Blue	**Yellow**
	*Serratia marcescens* 387	Red	Brown
	*Serratia marcescens* 1344	Red	Brown
	*Serratia odorifera* 1379	Whitish yellow	**Yellow**
	*Serratia plymuthica* 1398	**Yellow**	**Yellow**
	*Serratia rubidaea* 1354	Red	**Yellow**

Fungi	*Candida albicans* 110	Blue white	Brown
	*Candida albicans* 111	Blue white	Brown
	*Candida kefyr* 116	—	—
	*Candida krusei* 114	White	**Yellow**
	*Candida parapsilosis* 120	Blue white	Brown
	*Candida tropicalis* 651	White	**Yellow**
	*Cryptococcus neoformans* 105	—	—
	*Cryptococcus neoformans* 119	Blue white	Brown

Gram-positive rod	*Bacillus cereus* 77	—	—
	*Bacillus subtilis* 625	—	—
	*Bacillus subtilis* 626	—	—

Gram-positive cocci	*Enterococcus casseliflavus* 70	—	—
	*Enterococcus faecalis* 106	—	—
	*Enterococcus faecalis* 545	—	—
	*Enterococcus faecalis* 546	—	—
	*Enterococcus faecium* 547	—	—
	*Enterococcus faecium* 548	—	—
	*Enterococcus gallinarum* 69	—	—
	*Staphylococcus aureus* 75	—	—
	*Staphylococcus aureus* 89	—	—
	*Staphylococcus epidermidis* 58	—	—
	*Streptococcus oralis* 135	—	—
	*Streptococcus pneumoniae* 58	—	—
	*Streptococcus pyogenes* 538	—	—
	*Streptococcus sanguinis* 83	—	—

*1 Synonym of the type strain of *Pantoea agglomerans* American Type Culture Collection 27155.

*2 LOMAC: Lysine-Ornithine-Mannitol-Arginine-charcoal.

*3 No growth.

**Table 2:** Evaluation of semi-selective LOMAC agar medium for the cultivation of *Pantoea* spp. from environmental soils

Soil	LOMAC agar medium		Species identified by the biochemical test Api20E (No. of isolates tested)	% Positive predictive value	% Negative predictive value	% Overall agreement

	Total no. of colonies	No. of yellow colonies				
A	55	5	**Yellow colonies: *Pantoea* sp3 (5)** **Non-yellow colonies:** *Aeromonas hydrophila/caviae/sobria* (1), *Bordetella/Alcaligenes/Moraxella* spp. (2), *Enterobacter amnigenus* (3), *Ochrobacterium anthropi* (1), *Photobacterium damselae* (1), *Pseudomonas aeruginosa* (6), *Pseudomonas fluorescens/putida* (31), *Pseudomonas luteora* (3), *Pseudomonas oryzihabitans* (1), *Stenotrophomonas maltophilia* (1)	100% (5/5)	100% (50/50)	100% (55/55)
B	30	4	**Yellow colonies: *Pantoea* sp3 (1)**, *Citrobacter youngae* (2), *Leclercia adecarboxylate* (1), **Non-yellow colonies:** *Acinetobacter baumannii/calcoaceticus* (5), *Enterobacter cloacae* (10), *Hafnia alvei* (1), *Klebsiella pneumoniae* (1), *Pseudomonas aeruginosa* (1), *Pseudomonas fluorescens/putida* (8)	25% (1/4)	100% (26/26)	90% (27/30)
C	14	1	**Yellow colonies: *Pantoea* sp3 (1)** **Non-yellow colonies:** *Pseudomonas fluorescens/putida* (13)	100% (1/1)	100% (13/13)	100% (14/14)
	99	10	17 species	70% (7/10)	100% (89/89)	97% (96/99)

**Table 3:** *Pantoea* species isolated in 26 practical trials

Trial	Location	Sample	LOMAC agar medium			Species identified by the biochemical test Api20E (No. of isolates tested)

			Total no. of colonies	No. of yellow colonies	No. of yellow colonies selected for biochemical test Api20E*1	
Trial 1	Nagano C-1	Soil	39	24	7	***Pantoea* spp3 (1)**
Trial 2	Nagano A-1	Soil	19	0	0	None
Trial 3	Nagano B-1	Weed leaf (d*2)	209	48	8	***Pantoea* spp3 (7)**
		Weed root (d)	106	29	8	***Pantoea* spp3 (3)**, ***Pantoea* spp4 (1)**
		Soil	59	4	4	None
Trial 4	Nagano C-2	Soil	8	0	0	None
Trial 5	Nagano D-1	Soil	36	0	0	None
Trial 6	Nagano E-1	Soil	107	3	1	*Serratia rubidaea* (1)
Trial 7	Nagano F-1	Soil	54	0	0	None
Trial 8	Nagano G-1	Crops seed (d)	466	57	13	***Pantoea* spp3 (13)**
		Crops root (d)	888	49	7	***Pantoea* spp2 (6), *Pantoea* spp3 (1)**
		Soil	9	3	3	None
Trial 9	Nagano H-1	Soil	15	0	0	None
Trial 10	Nagano I-1	Soil	49	0	0	None
Trial 11	Nagano A-2	Soil	96	0	0	None
Trial 12	Nagano B-2	Soil	183	5	4	***Pantoea* spp3 (1)**, *Klebsiella ozaenae* (2)
Trial 13	Nagano A-3	Soil	15	3	1	None
		Weed leaf (d)	27	27	6	***Pantoea* spp2 (6)**
		Weed root (d)	7	0	0	None
Trial 14	Nagano B-3	Weed root (d)	75	6	4	***Pantoea* spp3 (1), *Pantoea* spp2 (1)**
		Soil	13	5	3	None
Trial 15	Nagano C-3	Weed root (d)	37	7	5	***Pantoea* spp2 (1)**, *Klebsiella ozaenae* (3)
		Weed leaf (d)	59	36	8	***Pantoea* spp3 (8)**
		Soil	34	2	2	None
Trial 16	Nagano D-2	Soil	15	0	0	None
Trial 17	Nagano E-2	Soil	175	4	4	None
Trial 18	Nagano F-2	Soil	40	4	3	***Pantoea* spp3 (1)**, *Klebsiella ozaenae* (1), *Leclercia adecarboxylate* (1)
Trial 19	Nagano G-2	Vegetable root (d)	59	43	4	None
		Soil	50	0	0	None
Trial 20	Nagano H-2	Forage vegetable (d)	410	336	11	***Pantoea* spp3 (7), *Pantoea* spp2 (3)**, *Rahnella aquatilis* (1)
		Soil	4	0	0	None
Trial 21	Fukuoka I-2	Fruit accessory (d)	36	36	4	***Pantoea* spp3 (4)**
		Soil	110	0	0	None
Trial 22	Fukuoka J	Soil	644	6	6	None
		Vegetable leaf (d)	1080	15	4	***Pantoea* spp1 (1), *Pantoea* spp3 (1)**, *Cronobacter* sp (1),
		Vegetable root (d)	472	8	6	***Pantoea* spp3 (2)**
Trial 23	Chiba K	Vegetable leaf (d)	90	6	5	***Pantoea* spp3 (5)**
		Vegetable root (d)	3	3	3	***Pantoea* spp3 (3)**
		Soil	95	0	0	None
Trial 24	Chiba L	Soil	41	4	2	***Pantoea* spp2 (2)**
		vegetable root (d)	0	0	0	None
Trial 25	Hokkaido M	Vegetable stem (m*3)	23	16	3	None
Trial 26	Nagano N	Vegetable stem (m)	32	32	3	***Pantoea* spp3 (2)**

		Total colony	5186	797	91	91

*1 All isolates produced a yellow pigment on TSA medium.

*2 d: dicot.

*3 m: monocot.
